# Spatial distribution characteristics of heavy metal(loid)s health risk in soil at scale on town level

**DOI:** 10.1038/s41598-022-20867-4

**Published:** 2022-11-10

**Authors:** Cang Gong, Haichuan Lu, Zhixiang Zhang, Liang Wang, Xiang Xia, Lihua Wang, Zhiyu Xiang, Linyang Shuai, Yang Ding, Yong Chen, Shunxiang Wang

**Affiliations:** Research Center of Applied Geology of China Geological Survey, Chengdu, China

**Keywords:** Environmental sciences, Environmental social sciences

## Abstract

In order to reveal the Spatial distribution characteristics of heavy metal(loid)s health risk in soil on the town-scale, 788 topsoil samples were collected from a town in the hinterland of Chengdu Plain, with 5 subordinate communities and 17 administrative villages as the research sub regions. The USEPA health risk assessment model was used to assess the health risks of heavy metal(loid)s Cd, Hg, As, Cu, Pb, Cr, Zn and Ni in the soil, the health risk analysis method system based on the geographic detector and the optimized rank-size theory model are used to clarify the spatial differentiation and risk level difference of health risk in the study area. The results showed that the average values of Cd, Hg, As, Pb, Cr, Cu, Ni and Zn in the soil of the study area were 0.221, 0.155, 9.76, 32.2, 91.9, 35.2, 37.1 and 108.8 mg/kg, respectively. The health risks of heavy metal(loid)s in soil to adults and children are generally within the acceptable range, but the maximum hazard index of children and the maximum non-carcinogenic risk value of Cr to children are 2.653303 and 1.213098 respectively, which were exceeding the acceptable range. The carcinogenic risk of Cr and As to adults and children and the carcinogenic risk of Cd to children are at 1 × 10^–4^ to 1 × 10^–6^, exceeding the 10^–6^ health threshold. The *q*-value range of heavy metal(loid)s health risk spatial differentiation of soil in the study area is 0.016–0.425. The spatial differentiation of non-carcinogenic risk of Hg, As, Ni, Pb, Cd and Cr and the spatial differentiation of carcinogenic risk of Cr and As are larger, which needs further attention. The strictly controlled area of heavy metal(loid)s health risk in the soil of the study area (R ≥ 1.1) is mainly concentrated in the central, western and northeast sub regions, and most sub regions belong to the safe utilization area (0.9 < R < 1.1). The health risk assessment of heavy metal(loid)s in soil on a town scale is of positive significance for enriching health risk research methods, measuring health risk levels at different scales, and formulating refined risk management and control strategies.

## Introduction

In the past decades, soil heavy metal(loid)s (HMs) pollution has become an important environmental problem because of its persistence, biological toxicity, carcinogenicity and refractory^1^. According to the China's national soil pollution survey in 2014, the total over standard rate of soil in China was 16.1%, and the proportions of slight, mild, moderate and severe pollution sites were 11.2%, 2.3%, 1.5% and 1.1%, respectively, the over standard rate of point for Cd, Hg, As, Cu, Pb, Cr, Zn and Ni were 7.0%, 1.6%, 2.7%, 2.1%, 1.5%, 1.1%, 0.9% and 4.8% respectively, the over standard rate of cultivated soil points was 19.4%, and the over standard points of inorganic pollutants accounted for 82.8% of all the over standard points. HMs pollution will change the physical and chemical properties of soil, damage the ecological environment, and even lead to disastrous environmental problems. Soil is the main medium for the transmission of HMs to water, atmosphere and organisms. HMs can be absorbed and accumulated by human body through direct inhalation, skin absorption or crop intake, affecting human health, and then leading to serious public health problems^2–4^. The excessive accumulation of HMs in soil has a great impact on human health and regional ecosystem balance. Therefore, in recent years, scholars at home and abroad have carried out research on soil HMs pollution and human health risks under different conditions such as natural conditions, industrial and mining industry and traffic development in different regions^5–13^. Mehr et al.^11^ reported that the health risk, bioavailability and distribution of HMs in agricultural soils of Kermanshah Province, west of Iran. Guo et al.^14^ found that the Cd is main polluting element in Xiong'an new district of China, and the non-carcinogenic and carcinogenic health risk hazards of soil HMs for adults are within the acceptable range, but only one soil sample has certain health risks for children. However, the current research methods are mainly based on the health risk assessment of soil HMs such as exposure parameters and exposure routes, the basic research unit focuses on the survey sample points or the spatial interpolation unit based on the survey sample points. The analysis and Research on the health risk of heavy metal(loid)s in agricultural soil based on the size of the town containing multiple survey sample points is less. At the same time, the relevant research methods and theoretical basis of the spatial feature analysis of health risk assessment need to be deepened.

Geographical detector^15^ as the detection of different scale variables space differentiation characteristics or exploratory spatial data analysis of the consistency between powerful tool, more intuitive, fast and effective measure of the contribution of each factor, there is no strong model assumption, solve the limitations of traditional methods in the analysis category variables^16,17^. It has been widely used in many fields such as underground^18^, land use^19^ and ecological vulnerability^20^. But the research content focuses on the detection of the correlation and interaction between variables, and there are few studies to directly measure the spatial differentiation characteristics of a single variable. Therefore, taking the HMs survey sample points as the number of units in the study area, the geographic detector is applied to the analysis of the spatial differentiation characteristics of soil HMs health risk, so as to explore and analyze the spatial differentiation characteristics, it not only enriches the practical application field of geographic detectors, but also provides early support for subsequent health risk level analysis^21^. At the same time, according to the evaluation results of the spatial differentiation characteristics of the health risk of HMs in soil, the rank-size theory model with the fractal characteristics of a certain thing in a certain time and space range is used to comprehensively evaluate the regional health risk rank containing the sample point position data of HMs in multiple soil, so as to characterize the comprehensive level of health risk, it can solve the problems of lack of research on regional comprehensive risk level measurement and less research on spatial characteristics^21^.

Therefore, this study takes a town contain 5 communities and 17 administrative villages in the heart of the Chengdu Plain as the research area, trying to embed the geographic detector and the rank-size theory model into the health risk analysis of soil HMs, analyze the regional overall health risk differentiation characteristics and the risk levels between sub-regions, and clarify the spatial differentiation characteristics and health risk level of regional comprehensive health risk. It is expected to provide reference for exploring and constructing cultivated land soil environmental management and control strategy from a multi-dimensional perspective, and further enrich the research methods of environmental health risk.

## Material and methods

### Study area

**Figure 1:**
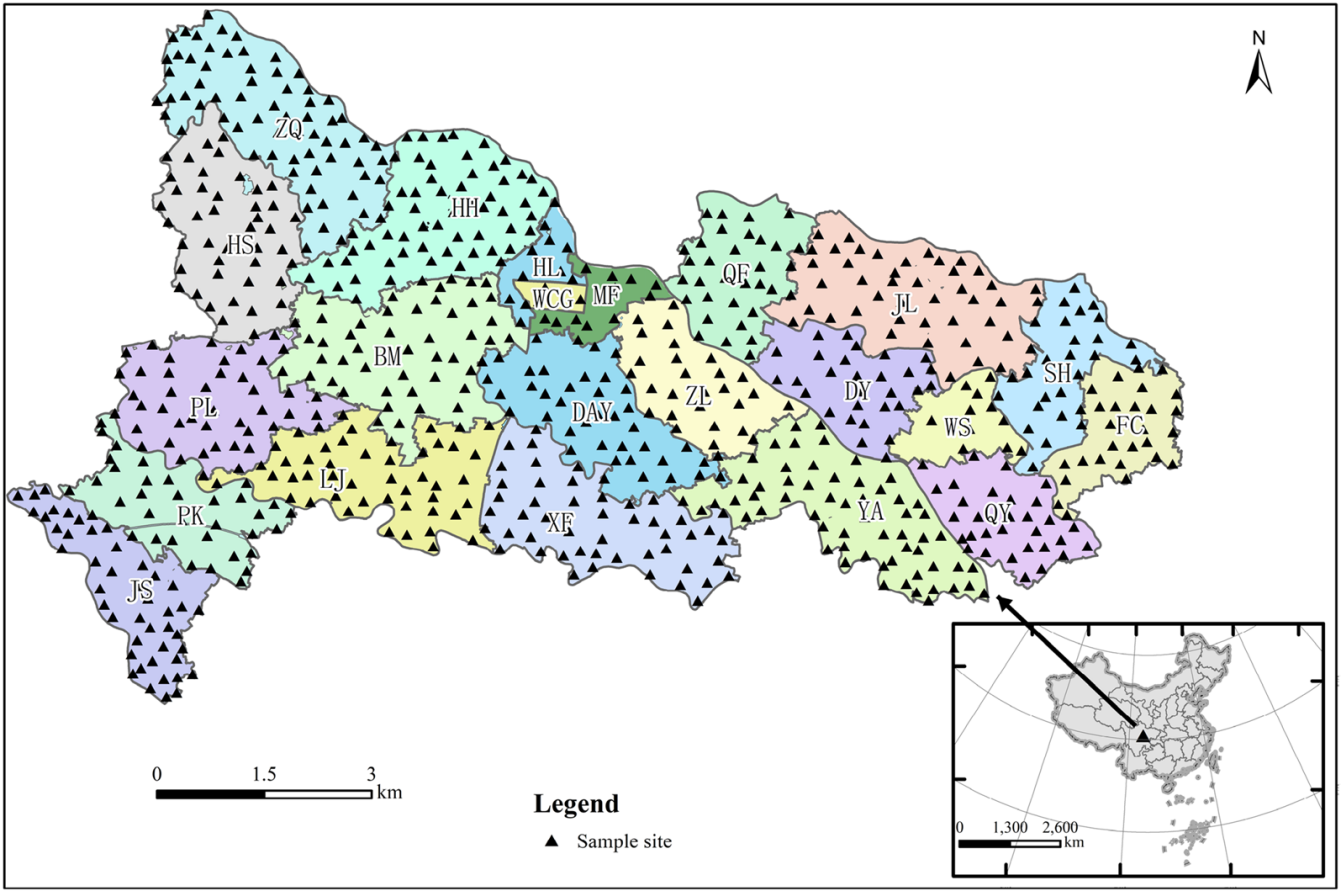
Locations of sub regions of study area and sampling sites (map were generated with software ArcMap10.8 http://www.esri.com/).

As shown in Fig. 1, the research area is located in the hinterland of Chengdu Plain in southwest China, about 40 km away from the urban area of Chengdu. It covers an area of 74.6 km^2^ and 95% of the cultivated land. It governs five communities including Wanshou (WS), Jinsha (JS), Hanlin (HL), Wenchangong (WCG) and Mingfeng (MF), and 17 administrative villages including Dayun (DAY), Liujia (LJ), Hengshan (HS), Xianfeng (XF), Huohua (HH), Zhanqi (ZQ), Zhulin (ZL), pingkang (PK), Pingle (PL), Baimu (BM), Yongan (YA), Qingyang (QY), Fuchang (FC), Shahe (SH), Jinning (JL), Diaoyu (DY) and Qianfu (QF). It has a subtropical monsoon humid monsoon climate with an average annual temperature of 15.7 °C, an average annual rainfall of 972 mm and an average annual sunshine time of 1280.9 h. The soil is fertile and belongs to purple alluvial soil. It is an important vegetable basket base in Chengdu, the largest hotbed chives production base in Southwest China, and the "hometown of hotbed chives" in China.

### Sample collection and measurement

Field sampling was performed in April 2021. The sample collection was done based on specification of land quality geochemical assessment (DZ/T 0295-2016), a total of 788 topsoil (0–20 cm) samples were collected. Among them, there are 13 in WS, 45 in JS, 12 in HL, 3 in WCG, 12 in MF, 40 in DAY, 44 in LJ, 42 in HS, 50 in XF, 56 in HH, 67 in ZQ, 27 in ZL, 36 in PK, 43 in PL, 51 in BM, 59 in YA, 31 in QY, 27 in FC, 26 in SH, 46 in JL, 27 in DY and 32 in QF. The sampling locations are shown in Fig. 1. At the same time, to improve the representativeness of the soil samples, five sub-samples were collected from a 20–50 m region around each sampling point by X-type sampling method and mixed into one sample, the sampling sites were located using a portable GPS. The samples were stored in sealed polyethylene bags until they were transported to the laboratory and were naturally air-dried for 1 week, removed other debris, and then sieved through a 10-mesh plastic sieve for later use.

In order to determine heavy metals contents of Cu, Pb, Zn, Cr, Ni and Cd, 0.1000 g soil samples were weighed accurately, placed into a PVC digestion vessel, and then digested with 10 ml HNO_3_-HCl-HClO_4_-HF (excellent grade). The concentrations of Cu, Pb, Zn, Ni and Cd were measured by inductively coupled plasma mass spectrometry (ICP-MS, NexION350X, USA)^22^, Cr were measured by inductively coupled plasma atomic emission spectrometry (ICP-AES, Avio500, USA). For As and Hg, 0.2500 g soil samples were weighed, placed into a tall beaker, and by water bath digestion with 10 ml aqua regia, the contents were measured by atomic fluorescence spectrophotometry (AFS, AFS8500, CHN)^23,24^. In the process of measurement, duplicate tests were carried out, and quality control procedures were conducted using state first-level standard materials (GSS5, 7–9, 20, 23, 24, 28). The Primary original qualified rate of all state first-level standard materials not lesser than 98%. The limit of detection (LOD) values for Cu, Pb, Zn, Ni, Cd, Cr, As and Hg were obtained to be 1, 2, 4, 2, 0.01, 5, 0.5 μg/g and 0.0003 μg/g, respectively. There were 64 samples for duplicate tests, the qualified rate of duplicate tests for HMs were 100%, except Hg was 93.8%.

### Health risk assessment

The human health risk assessment of HMs in soils is widely carried out using exposure evaluation. In this study, three exposure pathways associated with soil HMs were explored: ingestion (ADD_ing_), inhalation (ADD_inh_) and dermal contact (ADD_derm_). The estimated average daily intake of HMs (ADI, mg kg^−1^ day^−1^) via ADD_ing_, ADD_inh_ and ADD_derm_ for both adults and children was as follows^5,25^.

1 $${ADD}_{ing}=\frac{{C}_{i}\times IngR\times EF\times ED}{BW\times AT}\times {10}^{-6}$$

2 $${ADD}_{inh}=\frac{{C}_{i}\times InhR\times EF\times ED}{BW\times AT\times PEF}\times {10}^{-6}$$

3 $${ADD}_{derm}=\frac{{C}_{i}\times SA\times AF\times ABS\times EF\times ED}{BW\times AT\times PEF}\times {10}^{-6}$$

Detailed parameter values of IngR, InhR, EF, ED, BW, AT, PEF, SA, AF and ABS are listed in Table 1.

**Table 1:** Exposure parameters, reference dose (*RfD*) and slope factor (*SF*) of HMs.

Parameters	Value
IngR (ingestion rate of soil) (mg day^−1^)	100 (adults) and 200 (children)
InhR (inhalation rate) (m^3^/day)	14.5 (adults) and 7.5 (children)
EF (exposure frequency) (day/year)	350
ED (exposure duration) (year)	25 (adults) and 6 (children)
BW (average body weight) (kg)	56.8 (adults) and 15.9 (children)
AT (average exposure time) (days)	ED × 365 (non-carcinogenic risk) and 26,280 (carcinogenic risk)
SA (surface area of skin) (cm^2^)	5700 (adults) and 2800 (children)
AF (skin adherence factor) (mg/(cm^2^ h))	0.2
PEF (Emission factor) (m^3^/kg)	1.36 × 10^9^
ABS (dermal absorption factor) (unitless)	0.001
*RfD* for ingestion (unitless)	As (3.00 × 10^–4^), Cd (1.00 × 10^–3^), Cr (3.00 × 10^–3^), Cu (4.00 × 10^–2^), Hg (3.00 × 10^–4^), Ni (2.00 × 10^–2^), Pb (3.50 × 10^–4^) and Zn (3 × 10^–1^)
*RfD* for dermal absorption (unitless)	As (1.23 × 10^–4^), Cd (2.5 × 10^–5^), Cr (6 × 10^–5^), Cu (1.20 × 10^–2^), Hg (2.1 × 10^–5^), Ni (5.40 × 10^–3^), Pb (5.25 × 10^–5^) and Zn (6 × 10^–2^)
*RfD* for inhalation (unitless)	As (3.0 × 10^–4^), Cd (1.00 × 10^–5^), Cr (2.86 × 10^–5^), Cu (4.02 × 10^–2^), Hg (8.57 × 10^–5^), Ni (2.06 × 10^–3^), Pb (3.52 × 10^–5^) and Zn (3.0 × 10^–1^)
*SF* for ingestion (unitless)	As (1.50 × 10^0^), Cd (6.10 × 10^0^), Cr (5 × 10^–1^) and Pb (8.5 × 10^–3^)
*SF* for dermal absorption (unitless)	As (1.50 × 10^0^) and Cd (6.10 × 10^0^)
*SF* for inhalation (unitless)	As (1.51 × 10^1^), Cd (6.30 × 10^0^), Cr (4.20 × 10^1^), Ni (8.40 × 10^–1^) and Pb (4.20 × 10^–2^)

Hazard index (HI, unitless) and hazard quotient (HQ, unitless) are usually used to indicate the risk level of human exposure for non-carcinogenic contaminants. Carcinogenic risk (CR, unitless) is usually used to indicate the probability of inducing cancer when humans are exposed for carcinogenic contaminants. When the contaminants enter through multiple pathways of exposure, assuming that there is no antagonistic effect or cooperative effect between the contaminants, the HI for all exposure pathways and the CR can be calculated as follows^2^:

4 $$\mathrm{HI }= \sum \limits_{i=1}^{n}{HQ}_{i}=\sum \limits_{i=1}^{n}\frac{{ADD}_{i}}{{RfD}_{i}}$$

5 $$ {\text{CR }} = {\text{ ADD}} \times {\text{SF}} $$

where SF is the cancer slope factor of the HMs via ADD_ing_, ADD_inh_ and ADD_derm_ of soil particles (mg kg^−1^ day^−1^) and RfD is the reference dose of the HMs through ADD_ing_, ADD_inh_ and ADD_derm_ of soil particles (mg kg^−1^ day^−1^). Detailed values are listed in Table 1. Generally, HQ or HI is ≤ 1, it is considered that there are no significant risks of noncarcinogenic effects. For CR, the acceptable risk for regulatory purposes is in the range from 1.0 × 10^–6^ to 1.0 × 10^–4^.

### Geographical detector method

The geographic detector measures the contribution of independent variables to dependent variables by calculating the ratio of the sum of variance of respective variables to the sum of variance of dependent variables after classification. According to the results of health risk assessment, the differentiation and factor detection in geographic detectors are used to further explore the spatial differentiation characteristics of HMs health risks in different cultivated lands on the basis of health risk assessment. The specific evaluation model is as follows^15^:

6 $$q=1-\frac{\sum_{\mathrm{h}=1}^{L}{N}_{h}{\sigma }_{h}^{2}}{N{\sigma }^{2}}=1-\frac{\mathrm{SSW}}{\mathrm{SST}}$$

7 $$\mathrm{SSW}=\sum_{\mathrm{h}=1}^{L}{N}_{h}{\sigma }_{h}^{2}$$

8 $$\mathrm{SST}=N{\sigma }^{2}$$

where, *N*_*h*_ and N are the number of units in the study sub region *h* and the whole region respectively, and the number of survey samples is used as the basic unit of the study sub region; *σ*_*h*_^2^ and *σ*^2^ are the health risk variance of study sub region *h* and study area, respectively. SSW is the product of the health risk variance of each study sub region and the number of survey samples, and SST is the product of the total health risk variance of the whole region and the total number of survey samples. The *q* is the degree of spatial differentiation, which indicates the degree of spatial differentiation of health risks of heavy metal(loid)s in the study area. The *q* value range is [0,1], the greater the value of *q*, the higher the degree of spatial analysis of health risks, and vice versa.

Compare the average value of soil HMs health risk with the spatial diversity *q* of soil HMs health risk. If the average value of health risk at different survey points is high, but the *q* value is small, indicating that the spatial heterogeneity of cultivated land soil HMs within the study area is low, unified risk control can be carried out; If the average value of health risk is high and the *q* value is large, it indicates that the spatial difference of health risk within the study area is large, and it is necessary to formulate refined risk control measures according to local conditions^21^.

### Rank-size theory method

The rank-size theory was originally used to explore the relationship between urban rank and scale^26^. In this paper, the rank-size theory model optimized by Ji et al.^21^ was used, combined with the health risk assessment results of HMs at the sample points in the study sub area and the analysis results of geographic detectors, to obtain the health risk levels of HMs in cultivated land and soil in different sub areas in the study area. The specific calculation formula is as follows:

9 $${R}_{ij}=\frac{\sum_{j=1}^{n}\frac{{P}_{j}}{\overline{P}}}{n }$$

where, *R*_*ij*_ is the health risk level of the sub region, P_*j*_ is the health risk value of the survey sample point *j* in the study sub region *i*, *P* is the average health risk of the subregion, and *n* is the number of survey sample points in the sub region *i*. If *R*_*ij*_ > 0, indicates that the spatial distribution of HMs in the soil of sub region is characterized by aggregation, if *R*_*ij*_ = 0, means that the spatial distribution of HMs in the soil of sub region is characterized by balanced distribution, if *R*_*ij*_ < 0, means that the spatial distribution of HMs in the soil of sub region is characterized by dispersion^21,27,28^.

## Results and discussion

### Soil HMs concentrations

The soil HMs concentrations are summarized in Table 2. The mean concentration of Cd, Hg, As, Pb, Cr, Cu, Ni and Zn were 0.221, 0.155, 9.76, 32.2, 91.9, 35.2, 37.1 and 108.8 mg kg^−1^. Except Cd, the average concentration of Hg, As, Pb, Cr, Cu, Ni and Zn exceeded 93.8%, 7.1%, 6.3%, 17.8%, 25.3%, 10.7%, and 32.4% the soil background values for Chengdu, respectively, which indicates that HMs are enriched to a certain extent in soil. The CV of the HMs in the agricultural soils increased in the order Ni(10.1%), Cr(10.9%), Pb(15.4%), As(21.4%), Cd(31.1%), Zn(57.8%), Hg(58.1%) and Cu(59.9%). The exceptionally high variability of Cu, Hg and Zn indicates that these metal(loid)s differed greatly with respect to different sites, and the existence of abnormally high values is the main reason that the CV was high. It further indicates that Cu, Hg and Zn may be affected by external interference factors.

**Table 2:** Statistical summary of HMs concentrations (mg/kg) in soil.

Elements	Mean	S.D.	Median	Min	Max	CV%	Background values^a^	Threshold values^b^			
								A	B	C	D
Cd	0.221	0.069	0.210	0.082	0.83	31.05	0.25	0.3	0.3	0.3	0.6
Hg	0.155	0.090	0.130	0.022	0.88	58.13	0.08	1.3	1.8	2.4	3.4
As	9.76	2.08	9.43	4.17	18.0	21.35	9.11	40	40	30	25
Pb	32.2	5.0	32.3	19.8	90.3	15.42	30.3	70	90	120	170
Cr	91.9	10.0	92.3	61.7	264	10.87	78	150	150	200	250
Cu	35.2	21.1	34.4	18.5	607	59.93	28.1	50	50	100	100
Ni	37.1	3.7	37.2	23.6	56.6	10.07	33.5	60	70	100	190
Zn	108.8	62.9	106.0	55	1820	57.80	82.2	200	200	250	300

^a^The background values of soil metal(loid)s for Chengdu^29^.

^b^The risk screening values for soil contamination (GB 15618-2018) (MEEC, 2018), A: pH ≤ 5.5, B: 5.5 < pH ≤ 6.5, C: 6.5 < pH ≤ 7.5, D: pH > 7.5.

The mean concentration of all HMs in soil were below the risk screening values for soil contamination (GB 15618-2018), however, the results showed that in 76, 1, 1, 6 and 2 of sample sites the level of Cd, Pb, Cr, Cu and Zn exceeded the risk screening values, respectively. Due to the differences of exposure parameters and toxicity coefficients of different HMs and the superposition effect of health risks, the health risks of Cd, Hg, As, Pb, Cr, Cu, Ni and Zn need to be paid attention to.

### Health risk assessment

#### Overall health risk assessment

The adult non-carcinogenic risk value(HQa), child non-carcinogenic risk value(HQc), adult hazard index(HIa), child hazard index(HIc) of Cd, Hg, As, Pb, Cr, Cu, Ni and Zn, and adult carcinogenic risk value(CRa) and child carcinogenic risk value(CRc) of Cr, Cd, As, Ni and Pb in soil of the study area are calculated according to formulas (1)–(5), and the results are shown in Table 3.

**Table 3:** Statistics analysis for overall health risk of HMs of study aera.

Project	Max	Min	Average	Median	Mode	Variance
HQa-Cd	2.06E−03	2.03E−04	5.49E−04	5.20E−04	4.95E−04	1.71E−04
HQa-Hg	5.76E−03	1.44E−04	1.01E−03	8.51E−04	7.86E−04	5.91E−04
HQa-As	1.04E−01	2.41E−02	5.65E−02	5.45E−02	6.48E−02	1.21E−02
HQa-Pb	7.71E−02	1.69E−02	2.75E−02	2.76E−02	2.84E−02	4.27E−03
HQa-Cr	2.35E−01	5.49E−02	8.18E−02	8.21E−02	8.99E−02	8.96E−03
HQa-Cu	2.66E−02	8.11E−04	1.54E−03	1.51E−03	1.61E−03	9.33E−04
HQa-Ni	4.98E−03	2.08E−03	3.27E−03	3.28E−03	3.15E−03	3.30E−04
HQa-Zn	1.08E−02	3.27E−04	6.48E−04	6.31E−04	6.42E−04	3.78E−04
HIa	4.66E−01	9.95E−02	1.73E−01	1.72E−01	–	1.61E−02
HQc-Cd	1.12E−02	1.10E−03	2.98E−03	2.82E−03	2.69E−03	9.28E−04
HQc-Hg	3.68E−02	9.20E−04	6.46E−03	5.44E−03	5.02E−03	3.78E−03
HQc-As	7.29E−01	1.69E−01	3.95E−01	3.82E−01	4.53E−01	8.45E−02
HQc-Pb	3.70E−01	8.12E−02	1.32E−01	1.32E−01	1.36E−01	2.05E−02
HQc-Cr	1.21E+00	2.84E−01	4.22E−01	4.24E−01	4.64E−01	4.63E−02
HQc-Cu	1.85E−01	5.63E−03	1.07E−02	1.05E−02	1.12E−02	6.48E−03
HQc-Ni	3.45E−02	1.44E−02	2.26E−02	2.27E−02	2.18E−02	2.29E−03
HQc-Zn	7.42E−02	2.24E−03	4.44E−03	4.32E−03	4.40E−03	2.59E−03
HIc	2.65E+00	5.58E−01	9.97E−01	9.87E−01	/	1.00E−01
CRa-Cd	3.00E−06	2.97E−07	8.01E−07	7.60E−07	7.23E−07	2.50E−07
CRa-As	1.60E−05	3.71E−06	8.69E−06	8.39E−06	9.97E−06	1.86E−06
CRa-Pb	4.50E−07	9.87E−08	1.61E−07	1.61E−07	1.66E−07	2.50E−08
CRa-Cr	7.81E−05	1.82E−05	2.72E−05	2.73E−05	2.99E−05	2.98E−06
Cra-Ni	2.97E−09	1.24E−09	1.95E−09	1.96E−09	1.88E−09	1.97E−10
CRc-Cd	5.10E−06	5.04E−07	1.36E−06	1.29E−06	1.23E−06	4.24E−07
CRc-As	2.72E−05	6.31E−06	1.48E−05	1.43E−05	1.69E−05	3.16E−06
CRc-Pb	7.72E−07	1.69E−07	2.76E−07	2.76E−07	2.84E−07	4.28E−08
CRc-Cr	1.33E−04	3.11E−05	4.63E−05	4.65E−05	5.09E−05	5.07E−06
CRc-Ni	1.32E−09	5.49E−10	8.64E−10	8.68E−10	8.33E−10	8.74E−11

The average HQa for Cd, Hg, As, Pb, Cr, Cu, Ni and Zn in soil of the study area are 0.000549, 0.001012, 0.056452, 0.027544, 0.081777, 0.001543, 0.003267 and 0.000648, respectively, and the order is HQa-Cr > HQa-As > HQa-Pb > HQa-Ni > HQa-Cu > HQa-Zn > HQa-Cd > HQa-Hg. The max HQa value of eight HMs is HQa-Cr (0.234905), and the max and average of HIa are 0.466366 and 0.172813, respectively, which are less 1. It shows that the adult non carcinogenic risk of heavy metal(loid)s in soil in the study area is within the acceptable range. For the average HQc of Cd, Hg, As, Pb, Cr, Cu, Ni and Zn are0.002981, 0.006464, 0.395071, 0.132194, 0.422311, 0.010718, 0.022613 and 0.004440, respectively, and the order is HQa-Cr > HQa-As > HQa-Pb > HQa-Ni > HQa-Cu > HQa-Hg > HQa-Zn > HQa-Cd. The max HQc value of eight HMs is HQc-Cr (1.213098), and the max and average of HIc are 2.653303 and 0.996935, respectively, which are close to or greater than 1. It indicates that HMs in the soil of the study area have a non-carcinogenic risk for children. For average HI, the Cr, As and Pb were the largest contributors for both adults and children, accounting for 47.33% and 42.37%, 32.68% and 39.64%, 15.95% and 13.26%, respectively. Indicating that attention should be pain to the Cr, As and Pb elements due to their non-carcinogenic risk. Which was basically consistent with the research results of Bo et al.^2^ and Bao et al.^5^.

Since Hg, Cu and Zn in soil have no carcinogenic slope factor parameters, the carcinogenic risk of Cd, As, Pb, Cr and Ni are evaluated in study aera. The mean CRa for Cd, As, Pb, Cr and Ni are 0.0000008, 0.0000087, 0.0000002, 0.0000272 and 0.0000000, respectively, and the order is CRa-Cr > CRa-As > CRa-Cd > CRa-Pb > CRa-Ni. For the average CRc of Cd, As, Pb, Cr and Ni are 0.0000014, 0.0000148, 0.0000003, 0.0000463 and 0.0000000, respectively, and the order is the same as CRa of five HMs. The CRa-Cr, CRa-As, CRc-Cr, CRc-As and CRc-Cd obviously are in the range 1 × 10^–4^ from 1 × 10^–6^, suggesting the CR caused by HMs in the study area was acceptable on the whole, but it still exceeds the soil treatment threshold value 10^–6^. It is worth noting that the max CRc-Cr is 0.000132, which is significantly greater than 1 × 10^–4^, indicating that Cr in some areas of the study area has a high risk of carcinogenesis to children.

By comparing and analyzing the mean, maximum and minimum non carcinogenic risk and carcinogenic risk of HMs for adults and children in soil in the study area, it can be found that children's health risk index is higher than that of adults, and children are more vulnerable to soil heavy metal(loid)s, this result is basically consistent with most previous studies^30–32^. Which is mainly because the parameters of children's health risk assessment are set to be more sensitive than adults and more sensitive to environmental pollution^3,12,33^.

#### Sub regions health risk assessment

**Figure 2:**
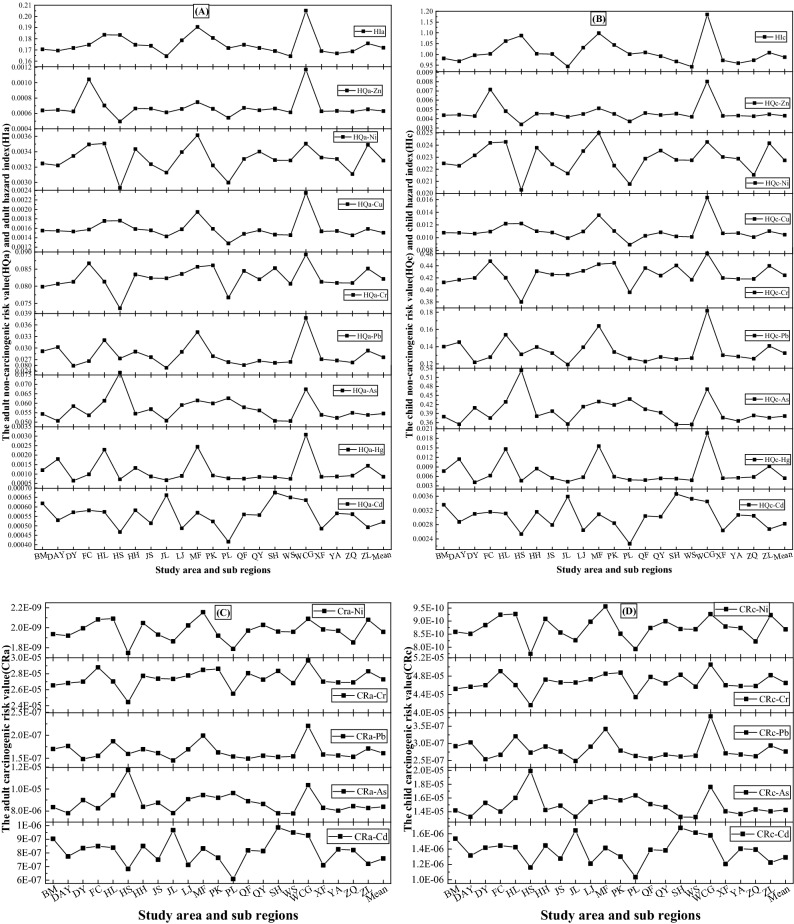
Health risks of HMS in the sub area of the study area.

The hazard index, non-carcinogenic risk and carcinogenic risk of HMs in soil of 22 sub areas of the study area to adults and children were shown in Fig. 2. Although the hazard index, non-carcinogenic risk and carcinogenic risk of the same heavy metal in different sub regions to children are higher than those of adults, the change trend of the health risk of the heavy metal to adults and children in different sub regions is the same, because the health risk changes with the content of HMs.

The health risks of HMs in soil in different sub regions are significantly different. In terms of non-carcinogenic risk (Fig. 2A,B), the maximum value of Cd and As appears in the sub region of SH, which are 0.000675 (Cd) and 0.07617 (As) for adults and 0.00367 (Cd) and 0.05055 (As) for children, the maximum HQa-Hg, HQa-Pb, HQa-Cr, HQa-Cu and HQa-Zn appears in WCG, which are 0.00308, 0.03781, 0.08925, 0.00235 and 0.00117 for adults and 0.01965, 0.18145, 0.46089, 0.01631 and 0.00802 for children, respectively. The max HQa-Ni value is found in MF, which is 0.00361 for adults and 0.02501 for children. Especially, the max HQa-Hg in WCG is 4.73 times of the min value HQa-Hg in HS. For the hazard index of HMs in 22 sub regions, the order is WCG > MF > HL > HS > PK > LJ > ZL > QF > HH > FC > JS > QY > DY > PL > BM > DAY > SH > XF > ZQ > YA > JL > WS, the max HI is 0.19058 of adult and 1.0986 of child. In terms of carcinogenic risk (Fig. 2C,D), the maximum CRa-Cd and CRa-As appears in the sub region of SH, the maximum Cra-Hg and Cra-Pb appears in WCG, and the max Cra-Ni is found sub regions MF.

### Spatial differentiation of HMs health risk based on geographic detector

The overall spatial differentiation characteristics of health risks of different HMs in soil in the study area were listed Table 4. It can be seen that the range of *q* value of spatial heterogeneity of health risks of HMs in soil in the study area is 0.016–0.425, and there are great differences in the spatial diversity of health risks of different HMs, which means that the health risks of heavy metal(loid)s in different soil within the scope of town size are quite different. Significantly, although the health risk variances of adults and children with HMs in soil in different study sub regions are different, the *q* values of non-carcinogenic risk and carcinogenic risk of adults and children with HMs in soil of different soil are the same, and the spatial differentiation characteristics are consistent. There is a significant difference with the conclusions drawn by Ji et al.^21^, they pointed out that the spatial differentiation *q* of health risk of HQ-Hg, HI and CR-Cr for adults is higher than that of children, and the *q* of health risk of HQ-Cr, HQ-Pb and CR-Cd for children was higher than that in adults. The reasons for the differences need to be further explored.

**Table 4:** Analysis of the spatial differentiation characteristics health risk of HMs based on geographic detector.

Project	HQa-Cd	HQa-Hg	HQa-As	HQa-Pb	HQa-Cr	HQa-Cu	HQa-Ni	HQa-Zn	HIa	HQc-Cd	HQc-Hg	HQc-As	HQc-Pb	HQc-Cr
*σ*_*BM*_^2^	3.84E−08	1.87E−07	8.08E−05	1.18E−05	4.59E−05	1.91E−07	1.01E−07	1.06E−08	2.05E−04	1.13E−06	7.65E−06	3.96E−03	2.72E−04	1.22E−03
*σ*_*DAY*_^2^	3.29E−08	6.88E−07	1.05E−04	1.84E−05	2.31E−05	4.11E−08	4.97E−08	7.05E−09	1.16E−04	9.71E−07	2.81E−05	5.17E−03	4.25E−04	6.16E−04
*σ*_*DY*_^2^	1.69E−08	3.75E−08	1.20E−04	9.14E−06	3.36E−05	3.00E−08	8.63E−08	4.30E−09	1.63E−04	4.99E−07	1.53E−06	5.87E−03	2.10E−04	8.97E−04
*σ*_*FC*_^2^	2.51E−08	1.91E−07	1.28E−04	9.88E−06	1.17E−04	4.99E−08	1.85E−07	3.70E−06	3.32E−04	7.41E−07	7.80E−06	6.28E−03	2.28E−04	3.11E−03
*σ*_*HL*_^2^	2.78E−08	6.97E−07	1.08E−04	5.36E−05	3.48E−05	1.42E−08	1.04E−07	6.43E−09	1.94E−04	8.19E−07	2.84E−05	5.30E−03	1.24E−03	9.29E−04
*σ*_*HS*_^2^	8.82E−08	5.95E−08	2.34E−04	1.05E−05	6.45E−05	1.51E−05	1.47E−07	6.67E−09	3.87E−04	2.60E−06	2.43E−06	1.15E−02	2.43E−04	1.72E−03
*σ*_*HH*_^2^	2.59E−08	2.87E−07	8.19E−05	1.21E−05	3.15E−05	3.41E−08	6.75E−08	6.09E−09	1.25E−04	7.64E−07	1.17E−05	4.01E−03	2.79E−04	8.40E−04
*σ*_*JS*_^2^	1.11E−08	2.53E−07	7.67E−05	4.91E−06	2.62E−05	1.67E−08	5.30E−08	2.12E−09	1.28E−04	3.28E−07	1.03E−05	3.76E−03	1.13E−04	6.98E−04
*σ*_*JL*_^2^	2.07E−08	4.01E−08	7.18E−05	8.55E−06	5.74E−05	2.10E−08	1.15E−07	8.99E−09	1.32E−04	6.11E−07	1.64E−06	3.52E−03	1.97E−04	1.53E−03
*σ*_*LJ*_^2^	8.88E−09	7.79E−08	8.31E−05	5.94E−05	2.11E−05	2.32E−08	4.69E−08	4.79E−09	2.34E−04	2.62E−07	3.18E−06	4.07E−03	1.37E−03	5.62E−04
*σ*_*MF*_^2^	3.44E−08	1.80E−06	1.18E−04	4.99E−05	1.70E−05	1.13E−07	2.82E−08	8.30E−09	9.52E−05	1.02E−06	7.36E−05	5.78E−03	1.15E−03	4.52E−04
*σ*_*PK*_^2^	2.29E−08	7.80E−08	8.48E−05	8.76E−06	6.56E−04	2.51E−07	5.69E−08	3.48E−09	7.19E−04	6.76E−07	3.18E−06	4.15E−03	2.02E−04	1.75E−02
*σ*_*PL*_^2^	1.14E−08	6.29E−08	2.07E−04	9.53E−06	5.38E−05	4.77E−08	1.87E−07	6.88E−09	2.87E−04	3.36E−07	2.57E−06	1.02E−02	2.20E−04	1.44E−03
*σ*_*QF*_^2^	1.54E−08	5.02E−08	1.23E−04	1.12E−05	4.98E−05	2.83E−08	8.29E−08	1.59E−08	2.26E−04	4.54E−07	2.05E−06	6.00E−03	2.58E−04	1.33E−03
*σ*_*QY*_^2^	2.04E−08	1.66E−07	1.10E−04	6.71E−06	6.44E−05	2.74E−08	9.85E−08	2.25E−09	2.19E−04	6.02E−07	6.76E−06	5.37E−03	1.55E−04	1.72E−03
*σ*_*SH*_^2^	3.14E−08	1.14E−07	6.82E−05	8.57E−06	2.56E−05	1.96E−08	1.43E−07	8.84E−09	1.57E−04	9.27E−07	4.66E−06	3.34E−03	1.97E−04	6.83E−04
*σ*_*WS*_^2^	7.06E−09	1.36E−07	7.07E−05	2.88E−06	2.52E−05	6.59E−09	5.95E−08	1.67E−09	1.29E−04	2.08E−07	5.53E−06	3.46E−03	6.64E−05	6.72E−04
*σ*_*WCG*_^2^	6.80E−08	1.26E−06	1.93E−05	1.01E−04	1.40E−04	7.16E−07	6.20E−08	5.15E−07	8.53E−04	2.00E−06	5.14E−05	9.43E−04	2.32E−03	3.73E−03
*σ*_*XF*_^2^	2.12E−08	8.87E−08	9.45E−05	1.85E−05	4.04E−05	1.66E−08	5.25E−08	2.60E−09	1.68E−04	6.24E−07	3.62E−06	4.63E−03	4.27E−04	1.08E−03
*σ*_*YA*_^2^	1.88E−08	1.04E−07	9.20E−05	9.57E−06	5.53E−05	3.95E−08	7.44E−08	3.02E−09	2.17E−04	5.56E−07	4.25E−06	4.51E−03	2.20E−04	1.48E−03
*σ*_*ZQ*_^2^	2.15E−08	1.16E−07	1.69E−04	8.51E−06	3.30E−05	1.09E−08	5.01E−08	2.93E−09	2.13E−04	6.35E−07	4.75E−06	8.29E−03	1.96E−04	8.79E−04
*σ*_*ZL*_^2^	2.07E−08	3.94E−07	9.05E−05	1.09E−05	2.22E−05	1.99E−08	2.12E−08	2.05E−09	1.37E−04	6.10E−07	1.61E−05	4.43E−03	2.52E−04	5.93E−04
*σ*^2^	2.92E−08	3.49E−07	1.46E−04	1.83E−05	8.03E−05	8.70E−07	1.09E−07	1.43E−07	2.58E−04	8.61E−07	1.43E−05	7.14E−03	4.21E−04	2.14E−03
SSW/SST	0.864	0.575	0.770	0.809	0.887	0.984	0.780	0.939	0.869	0.864	0.575	0.770	0.809	0.887
*q*	0.136	0.425	0.230	0.191	0.113	0.016	0.220	0.061	0.131	0.136	0.425	0.230	0.191	0.113

Project	HQc-Cu	HQc-Ni	HQc-Zn	HIc	CRa-Cd	CRa-As	CRa-Pb	CRa-Cr	CRa-Ni	CRc-Cd	CRc-As	CRc-Pb	CRc-Cr	CRc-Ni
*σ*_*BM*_^2^	9.21E−06	4.84E−06	4.96E−07	7.66E−03	8.19E−14	1.91E−12	4.02E−16	5.07E−12	3.59E−20	2.37E−13	5.52E−12	1.18E−15	1.47E−11	7.06E−21
*σ*_*DAY*_^2^	1.98E−06	2.38E−06	3.31E−07	4.81E−03	7.02E−14	2.50E−12	6.28E−16	2.55E−12	1.77E−20	2.03E−13	7.21E−12	1.85E−15	7.40E−12	3.48E−21
*σ*_*DY*_^2^	1.45E−06	4.14E−06	2.02E−07	6.79E−03	3.61E−14	2.84E−12	3.11E−16	3.72E−12	3.07E−20	1.04E−13	8.20E−12	9.15E−16	1.08E−11	6.03E−21
*σ*_*FC*_^2^	2.41E−06	8.86E−06	1.74E−04	1.24E−02	5.36E−14	3.04E−12	3.37E−16	1.29E−11	6.57E−20	1.55E−13	8.77E−12	9.89E−16	3.73E−11	1.29E−20
*σ*_*HL*_^2^	6.86E−07	4.98E−06	3.02E−07	7.17E−03	5.93E−14	2.56E−12	1.83E−15	3.85E−12	3.69E−20	1.71E−13	7.40E−12	5.37E−15	1.12E−11	7.26E−21
*σ*_*HS*_^2^	7.29E−04	7.06E−06	3.13E−07	1.58E−02	1.88E−13	5.54E−12	3.59E−16	7.12E−12	5.24E−20	5.44E−13	1.60E−11	1.05E−15	2.07E−11	1.03E−20
*σ*_*HH*_^2^	1.65E−06	3.23E−06	2.86E−07	4.88E−03	5.53E−14	1.94E−12	4.13E−16	3.48E−12	2.40E−20	1.60E−13	5.60E−12	1.21E−15	1.01E−11	4.72E−21
*σ*_*JS*_^2^	8.07E−07	2.54E−06	9.97E−08	5.20E−03	2.37E−14	1.82E−12	1.67E−16	2.89E−12	1.88E−20	6.86E−14	5.24E−12	4.91E−16	8.38E−12	3.71E−21
*σ*_*JL*_^2^	1.01E−06	5.49E−06	4.22E−07	4.97E−03	4.42E−14	1.70E−12	2.91E−16	6.34E−12	4.07E−20	1.28E−13	4.91E−12	8.55E−16	1.84E−11	8.01E−21
*σ*_*LJ*_^2^	1.12E−06	2.25E−06	2.25E−07	8.23E−03	1.90E−14	1.97E−12	2.02E−15	2.33E−12	1.67E−20	5.48E−14	5.68E−12	5.94E−15	6.75E−12	3.28E−21
*σ*_*MF*_^2^	5.45E−06	1.35E−06	3.90E−07	4.38E−03	7.35E−14	2.80E−12	1.70E−15	1.87E−12	1.00E−20	2.12E−13	8.07E−12	4.99E−15	5.44E−12	1.97E−21
*σ*_*PK*_^2^	1.21E−05	2.73E−06	1.63E−07	2.12E−02	4.89E−14	2.01E−12	2.98E−16	7.24E−11	2.02E−20	1.41E−13	5.79E−12	8.76E−16	2.10E−10	3.98E−21
*σ*_*PL*_^2^	2.30E−06	8.96E−06	3.23E−07	1.22E−02	2.43E−14	4.91E−12	3.25E−16	5.94E−12	6.65E−20	7.02E−14	1.42E−11	9.54E−16	1.72E−11	1.31E−20
*σ*_*QF*_^2^	1.37E−06	3.97E−06	7.49E−07	8.95E−03	3.28E−14	2.90E−12	3.81E−16	5.50E−12	2.95E−20	9.49E−14	8.38E−12	1.12E−15	1.60E−11	5.80E−21
*σ*_*QY*_^2^	1.32E−06	4.72E−06	1.06E−07	8.43E−03	4.35E−14	2.60E−12	2.29E−16	7.11E−12	3.50E−20	1.26E−13	7.50E−12	6.72E−16	2.06E−11	6.89E−21
*σ*_*SH*_^2^	9.46E−07	6.83E−06	4.15E−07	6.05E−03	6.71E−14	1.62E−12	2.92E−16	2.83E−12	5.06E−20	1.94E−13	4.66E−12	8.58E−16	8.20E−12	9.96E−21
*σ*_*WS*_^2^	3.18E−07	2.85E−06	7.84E−08	5.36E−03	1.51E−14	1.67E−12	9.82E−17	2.78E−12	2.11E−20	4.36E−14	4.83E−12	2.89E−16	8.08E−12	4.16E−21
*σ*_*WCG*_^2^	3.45E−05	2.97E−06	2.42E−05	2.54E−02	1.45E−13	4.56E−13	3.43E−15	1.54E−11	2.20E−20	4.19E−13	1.32E−12	1.01E−14	4.48E−11	4.34E−21
*σ*_*XF*_^2^	8.01E−07	2.52E−06	1.22E−07	6.42E−03	4.52E−14	2.24E−12	6.31E−16	4.46E−12	1.87E−20	1.31E−13	6.46E−12	1.85E−15	1.29E−11	3.67E−21
*σ*_*YA*_^2^	1.91E−06	3.56E−06	1.42E−07	8.06E−03	4.02E−14	2.18E−12	3.26E−16	6.11E−12	2.64E−20	1.16E−13	6.29E−12	9.58E−16	1.77E−11	5.20E−21
*σ*_*ZQ*_^2^	5.28E−07	2.40E−06	1.38E−07	9.32E−03	4.59E−14	4.01E−12	2.90E−16	3.64E−12	1.78E−20	1.33E−13	1.16E−11	8.52E−16	1.06E−11	3.50E−21
*σ*_*ZL*_^2^	9.59E−07	1.02E−06	9.64E−08	5.71E−03	4.41E−14	2.14E−12	3.73E−16	2.46E−12	7.55E−21	1.27E−13	6.19E−12	1.10E−15	7.13E−12	1.48E−21
*σ*^2^	4.20E−05	5.23E−06	6.70E−06	9.99E−03	6.23E−14	3.45E−12	6.23E−16	8.87E−12	3.88E−20	1.80E−13	9.96E−12	1.83E−15	2.57E−11	7.63E−21
SSW/SST	0.984	0.780	0.939	0.852	0.864	0.770	0.809	0.887	0.780	0.864	0.770	0.809	0.887	0.780
*q*	0.016	0.220	0.061	0.148	0.136	0.230	0.191	0.113	0.220	0.136	0.230	0.191	0.113	0.220

The *q* value of non-carcinogenic risk of HMs in soil for adults and children is ranked as HQ-Hg > HQ-As > HQ-Ni > HQ-Pb > HQ-Cd > HQ-Cr > HQ-Zn > HQ-Cu. The spatial differentiation of HQ-Hg is the largest, with *q* value of 0.425. The *q* value of non-carcinogenic risk of other HMs except Cr and Zn are greater than 0.1, indicating that the spatial differentiation of non-carcinogenic risk of Hg, As, Ni, Pb, Cd and Cr within the size range of the town is large, and the distribution of health risk among regions is quite different, it needs to be focused.

The *q* value of adult and child carcinogenic risk of Cd, As, Pb, Cr and Ni in soil is greater than 0.1, and the order is CR-As > CR-Ni > CR-Pb > CR-Cd > CR-Cr. The spatial differentiation of CR-As is the largest, with *q* value of 0.230, while the spatial differentiation of CR-Cr is the smallest, with *q* value of 0.113, indicating that Cd, As, Pb, Cr and Ni have obvious spatial differentiation characteristics, but different strengths. Combining the results of geographical detectors with the results of health risk analysis (Table 3), it is found that the heavy metal Cr with the greatest risk of carcinogenesis has the lowest spatial differentiation, which means that the spatial differentiation of soil heavy metal Cr in different sub regions of the study area is weak; the element As with higher risk of carcinogenesis, its spatial differentiation is the largest, indicating that the spatial distribution of As in soil of different sub regions is very different; heavy metal Ni has the lowest carcinogenic risk, but its spatial diversity is second only to As. Therefore, different measures should be taken according to different heavy metal elements in risk management and control. For example, the health risk level of the whole study area should be taken into account in risk management and control of Cr, Pb and Cd, while the difference in health risk level between sub regions should be further analyzed in risk management and control of As and Ni.

### Level of HMs health risk based on rank-size theory method

The rank-size distribution can effectively reflect the clustering characteristics of variables^34^. The results of health risk level of HMs based on rank-size theory method in soil of 22 sub regions in the study area are shown in Table 5. The non-carcinogenic risk value R of Cd ranges from 0.76 to 1.23, it has obvious aggregation characteristics in 14 sub regions such as BM, Dy, FC, FC, HL, HH, JL, MF, QF, QY, SH, WS, WCG, YA and ZQ, and obvious dispersion distribution characteristics in the other 8 sub regions. The R value of HQ-Hg ranges from 0.64 to 3.04, it has obvious aggregation characteristics in 7 sub regions such as BM, DAY, HL, HH, MF, WCG and ZL, especially, the R value in HL, MF and WCG are 2.26, 2.41 and 3.04, respectively, showing very strong aggregation characteristics. The R value of HQ-As has obvious aggregation characteristics in 10 sub regions such as DY, HL, HS, JS, LJ, MF, PK, PL, QF and WCG; R value of HQ-Pb has obvious accumulation characteristics in BM, DAY, HL, HH, JS, MF, PK, WCG and ZL; R value of HQ-Cr has obvious accumulation characteristics in FC, HH and JS, JL, LJ, MF, PK, QF, SH, WCG and ZL; R value of HQ- Cu in sub regions of BM, FC, HL, HS, HH, JS, LJ, MF, PK, QY, WCG and ZL, R value of HQ- Ni in DY, FC, HL, HH, LJ, MF, QF, QY, SH, WS, WCG, XF, YA and ZL, R value of HQ-Zn in FC, HL, HH, JS, LJ, MF, PK, QF, SH, WCG and ZL are higher than 1. The R value of hazard index ranges from 0.95 to 1.19, which in FC, HL, HS, HH, JS, LJ, MF, PK, QF, WCG and ZL is higher than 1. The R value of carcinogenic risk of Cd, As, Pb, Cr and Ni ranges from 0.76–1.23, 0.90–1.35, 0.90–1.37, 0.90–1.09 and 0.90–1.11, respectively, which in 14, 10, 9, 11 and 14 sub regions higher than 1, respectively, showing the characteristics of accumulation.

**Table 5:** Human health risk level of HMs in sub aera soil based on the optimized rank-scale theory model.

Project	HQ-Cd	HQ-Hg	HQ-As	HQ-Pb	HQ-Cr	HQ-Cu	HQ-Ni	HQ-Zn	HI	CR-Cd	CR-As	CR-Pb	CR-Cr	CR-Ni
R_-BM_	1.13	1.18	0.97	1.06	0.98	1.01	1.00	0.99	0.99	1.12	0.97	1.06	0.98	1.00
R_*-DAY*_	0.97	1.77	0.90	1.10	0.99	1.00	0.99	1.00	0.98	0.97	0.90	1.10	0.99	0.99
R_*-DY*_	1.04	0.64	1.03	0.92	0.99	0.99	1.02	0.97	0.99	1.04	1.03	0.92	0.99	1.02
R_*-FC*_	1.06	0.97	0.95	0.97	1.06	1.02	1.07	1.61	1.01	1.06	0.95	0.97	1.06	1.07
R_*-HL*_	1.04	2.26	1.09	1.16	0.99	1.14	1.07	1.08	1.06	1.04	1.09	1.16	0.99	1.07
R_*-HS*_	0.85	0.71	1.35	0.99	0.90	1.14	0.90	0.76	1.06	0.85	1.35	0.99	0.90	0.90
R_*-HH*_	1.06	1.30	0.97	1.06	1.02	1.03	1.05	1.02	1.01	1.06	0.97	1.06	1.02	1.05
R_*-JS*_	0.94	0.86	1.01	1.00	1.01	1.01	0.99	1.02	1.01	0.94	1.01	1.00	1.01	0.99
R_*-JL*_	1.21	0.67	0.90	0.90	1.01	0.93	0.96	0.95	0.95	1.21	0.90	0.90	1.01	0.96
R_*-LJ*_	0.89	0.89	1.04	1.05	1.02	1.02	1.04	1.02	1.03	0.89	1.04	1.05	1.02	1.04
R_*-MF*_	1.04	2.41	1.09	1.24	1.05	1.26	1.11	1.15	1.10	1.04	1.09	1.24	1.05	1.11
R_*-PK*_	0.95	0.91	1.06	1.01	1.05	1.03	0.99	1.02	1.05	0.95	1.06	1.01	1.05	0.99
R_*-PL*_	0.76	0.76	1.11	0.96	0.94	0.83	0.92	0.84	0.99	0.76	1.11	0.96	0.94	0.92
R_*-QF*_	1.02	0.75	1.02	0.93	1.03	0.96	1.01	1.04	1.01	1.02	1.02	0.93	1.03	1.01
R_*-QY*_	1.01	0.83	0.99	0.97	1.00	1.01	1.04	0.99	0.99	1.01	0.99	0.97	1.00	1.04
R_*-SH*_	1.23	0.81	0.90	0.95	1.04	0.95	1.01	1.02	0.98	1.23	0.90	0.95	1.04	1.01
R_*-WS*_	1.18	0.74	0.90	0.96	0.99	0.94	1.01	0.95	0.95	1.18	0.90	0.96	0.99	1.01
R_*-WCG*_	1.16	3.04	1.19	1.37	1.09	1.52	1.07	1.81	1.19	1.16	1.19	1.37	1.09	1.07
R_*-XF*_	0.88	0.84	0.95	0.98	0.99	1.00	1.02	0.97	0.98	0.88	0.95	0.98	0.99	1.02
R_*-YA*_	1.03	0.86	0.93	0.97	0.99	1.00	1.01	0.98	0.97	1.03	0.93	0.97	0.99	1.01
R_*-ZQ*_	1.02	0.90	0.97	0.95	0.99	0.94	0.95	0.96	0.98	1.02	0.97	0.95	0.99	0.95
R_*-ZL*_	0.90	1.42	0.95	1.06	1.04	1.03	1.07	1.01	1.02	0.90	0.95	1.06	1.04	1.07

**Figure 3:**
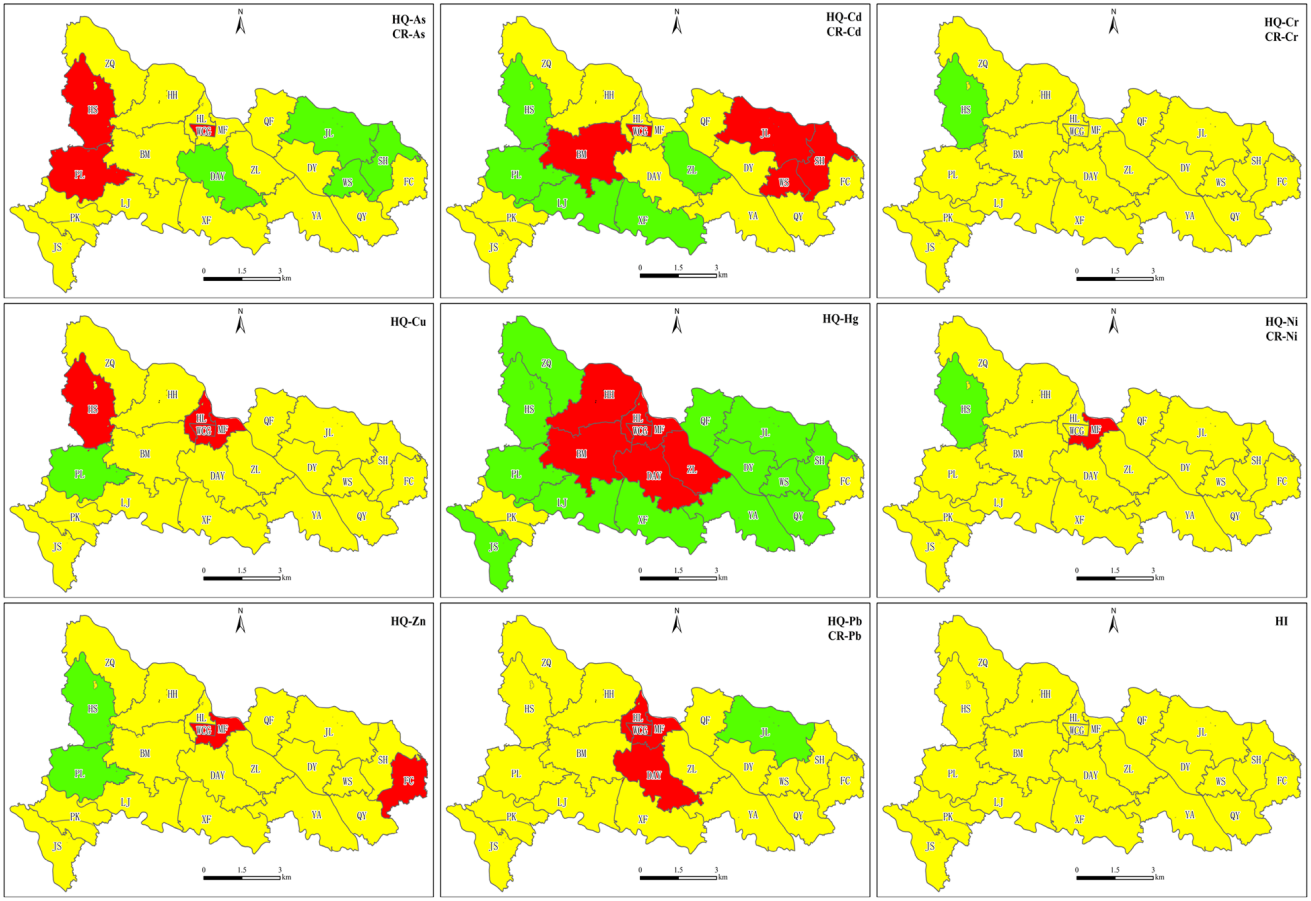
Health risk level of HMs in soil of different sub regions. (Green, yellow and red indicate low-risk level, medium-risk level and high-risk level respectively).

According to the "Action Plan on Prevention and Control of Soil Pollution"^35^, the soil environmental quality should be divided into priority protection areas, safe utilization areas and strict control areas for zoning control. Referring to the grading methods of Ji et al.^21^ and Lu et al.^36^, the health risk level of soil HMs in the sub region of the study area is divided into three grades: low-risk level, medium-risk level and high-risk level according to 0.9 ≤ , 0.9–1.1 and ≥ 1.1 of R value, and the results are visually displayed in Fig. 3.

It can be seen from the figure that the areas with R ≥ 1.1 of As are mainly concentrated in HS and PL in the southwest and WCG in the middle of the study area, these areas should be paid attention to as strict control areas during health risk zoning control. The areas with R ≤ of As are concentrated in DAY in the middle and JL, SH and WS in the northeast, these areas should be used as priority protection areas for health risk zoning control, and the R of other sub areas are between 0.9 and 1.1, it should be used as a safe utilization area for health risk zoning control. The strict control areas of Cd are distributed in WCG and BM in the middle and JL, SH and WS in the Northeast; the strict control areas of Cu are concentrated in WCG, MF and HL in the middle and HS in the West; the strict control areas of Hg are concentrated in WCG, MF, HL, HH, BM, DAY and ZL in the middle; the strict control area of Ni is only concentrated in MF in the middle; the strict control areas of Zn are concentrated in WCG and MF in the middle and FC in the East; the strict control areas of Pb are concentrated in HL, WCG, MF and DAY in the middle; Cr and hazard index are not strictly controlled areas in the study area. In general, the strictly controlled areas of HMs Health risks in soil of the study area are mainly concentrated in the middle and In a few sub regions in the West and northeast. Except for Hg and Cd, the priority protection areas of HMs health risks are only sporadically distributed in individual sub areas, and the health risks of most sub areas belong to safe utilization areas. For areas with strict control over health risks, measures should be taken to effectively reduce the health risks of heavy metal(loid)s in soil, and measures should be taken to ensure that the health risks of heavy metal(loid)s in soil do not increase in priority protection areas and safe utilization areas.

## Conclusion

The average concentration of all HMs in the soil of the study area was lower than the screening value of soil pollution risk. The adult non-carcinogenic risk level and hazard index of HMs in soil are low, and the maximum and average values of HQa-Cr, HQa-As, HQa-Pb, HQa-Ni, HQa-Cu, HQa-Zn, HQa-Cd and HQa-Hg are less than 1. Children have higher non-carcinogenic risk. The maximum value of HQc-Cr and the maximum and average values of HIc are close to or greater than 1. CRa-Cr, CRa-As, CRc-Cr, CRc-As and CRc-Cd are at 1 × 10^–4^ to 1 × 10^–6^, the maximum CRc-Cr is 0.000132, significantly greater than 1 × 10^–4^. Children have a higher risk of cancer. The hazard index, non-carcinogenic risk and carcinogenic risk of HMs in soil of different sub regions are higher than that of adults. The change trend of health risk of HMs in soil of different sub regions to adults and children is the same, and the health risk of HMs in soil of different sub regions is significantly different. The q value range of soil HMs health risk spatial differentiation is 0.016–0.425, the q value of soil HMs non-carcinogenic risk is HQ-Hg > HQ-As > HQ-Ni > HQ-Pb > HQ-Cd > HQ-Cr > HQ-Zn > HQ-Cu, the q value of carcinogenic risk is CR-As > CR-Ni > CR-Pb > CR-Cd > CR-Cr, the Cr with the highest carcinogenic risk has the lowest spatial diversity, while As with the higher carcinogenic risk has the highest spatial differentiation. The strict control area of HMs health risk in the soil of the study area is mainly concentrated in the central, western and northeast sub areas, the priority protection areas are only scattered in the sub areas, and most sub areas belong to safe utilization areas. Areas where health risks are strictly controlled should be given special attention. Due to the limitations of research data and the complexity of health risks, the research results may be lower than the actual situation. In the next research, we can further increase the research on health risk assessment of agricultural products, build a soil agricultural product health risk research system, and then lay a foundation for the overall management of various health risk characteristic factors. The research scale can be further extended to provincial and national scales, and then provide the basis for the implementation of health risk control measures of relevant national macro control strategies.

## Data Availability

The datasets used and/or analyzed during the current study are available from the corresponding author on reasonable request.
